# Editorial: Role of Bifidobacteria in Human and Animal Health and Biotechnological Applications

**DOI:** 10.3389/fmicb.2021.785664

**Published:** 2021-11-08

**Authors:** Maria Esteban-Torres, Lorena Ruiz, Gabriele Andrea Lugli, Marco Ventura, Abelardo Margolles, Douwe van Sinderen

**Affiliations:** ^1^School of Microbiology, APC Microbiome Ireland, University College Cork, Cork, Ireland; ^2^Department of Microbiology and Biochemistry, Institute of Dairy Products of Asturias, IPLA-CSIC, Villaviciosa, Spain; ^3^Functionality and Ecology of Beneficial Microbes (MicroHealth) Group, Instituto de Investigación Sanitaria del Principado de Asturias, Oviedo, Spain; ^4^Laboratory of Probiogenomics, Department of Chemistry, Life Sciences and Environmental Sustainability, University of Parma, Parma, Italy; ^5^Microbiome Research Hub, University of Parma, Parma, Italy

**Keywords:** probiotics, bifidobacteria, gut microbiome, prebiotics, biotechnology

The gut microbiota is a dynamic community playing a key role in maintaining and supporting host health (Zheng et al., 2020). The interactions between the gut microbiota and the host are complex and perturbations by different causes may result in a variety of diseases including infections, inflammatory bowel disease, metabolic syndrome, neurodegenerative disorders and malignancy (Zheng et al., 2020). In humans, members of *Bifidobacterium* genus are among the most abundant colonizers of the gut of healthy infants (Milani et al., 2017) and persist throughout adulthood at lower relative abundance with a further reduction in the elderly (Arboleya et al., 2016).

Certain bifidobacterial strains with purported health-promoting properties in the host are being commonly used as probiotics. These benefits include protection against pathogens, modulation of the host immune system, provision of nutrients and vitamins, among various other reported beneficial activities (O'Callaghan and van Sinderen, 2016; Wong et al., 2020). Based on these findings, it seems clear that the complex interactions between human host and bifidobacteria play a key role in health and disease.

The present Research Topic entitled “*Role of Bifidobacteria in Human and Animal Health and Biotechnological Applications*” is comprised of 11 articles. These scientific articles expand our knowledge on the interactions of bifidobacteria (and their metabolites) with its host, and their application as probiotics to improve human and animal health. Here, we summarize some of the scientific highlights of the articles published in this special issue.

Bifidobacteria metabolize a variety of complex carbohydrates that through their metabolic end products also provide nutrients to the host. This remarkable metabolic behavior reflects the importance of carbohydrate utilization as part of bifidobacterial gut colonization and persistence. The current knowledge of bifidobacterial carbohydrate metabolism is reviewed, with a focus on plant poly-/oligosaccharide degradation (Kelly et al.). Many so-called non-digestible glycans specifically stimulate growth of particular bifidobacterial strains and/or species. Among them, galacto-oligosaccharides (GOS) are widely used as prebiotics in infant nutrition. Ambrogi et al. showed that the enzyme BgaE, from *Bifidobacterium longum* subsp. *longum* NCIMB 8809, is suitable for *in vitro* GOS synthesis using lactose as the starting substrate. This work highlights the potential of bifidobacterial enzymes to be exploited for the development of dietary prebiotics.

Another feature of bifidobacterial metabolism with potentially important health implications is the *in situ* production of vitamins. Solopova et al. applied comparative genomics and phylogenomic analysis to investigate the acquisition and distribution of riboflavin (vitamin B2) biosynthesis-associated genes across the genus *Bifidobacterium*. The authors generated spontaneous riboflavin-overproducing variants of *Bifidobacterium longum* subsp. *infantis* ATCC 15697, which were also shown to increase vitamin B_2_ concentration in a fecal fermentation system, thereby providing promising data for application of this isolate as a functional food ingredient.

Commensal bacteria colonize the gut and by doing so offer protection against pathogens. Mechanisms of gastrointestinal protection by probiotic bacteria against infection involve modulation of intestinal epithelial barrier function. The application of trans-epithelial electrical resistance (TEER) has been used by Yuan et al. to evaluate the effect of bifidobacteria on intestinal epithelial layers when damaged by pathogenic *Escherichia coli*.

The production of extracellular layers, such as exopolysaccharide (EPS), by certain bifidobacteria taxa, allows them to overcome gastrointestinal challenges and to persist for longer periods in the gut (Hidalgo-Cantabrana et al., 2014). EPS produced by bacteria with probiotic traits has been associated with protective immunomodulatory effects (Delgado et al., 2020). In this context, some of the mechanisms underpinning the involvement of bifidobacterial EPS in immune cell response were investigated in more detail by Hickey et al.

Alterations in gut bacteria and their metabolites on several inflammatory and immune processes have been associated with carcinogenesis and tumor etiology (Fong et al., 2020). The current development of microbiome-based therapies has focused on the role of particular bifidobacterial species in cancer immunotherapy as reviewed here (Longhi et al.).

Probiotics are believed to become a potent tool to modify the composition of the gut microbiome and to benefit host health in multiple ways. As part of this Research Topic, several papers discuss the use of bifidobacterial strains as probiotics. For example, the use of *Bifidobacterium bifidum* JCM 1254 to treat antibiotic-induced dysbiosis was studied (Ojima et al.). The administration of the bifidobacterial strain resulted in the reduction of gut inflammation in mice without recovering gut microbiome diversity. This positive effect in the gut only occurs when proinflammatory species-induced gut inflammation (Ojima et al.).

To exert a probiotic effect, a bacterial strain may need to colonize and persist in the host. In the clinical trial carried out by Horigome et al. in low birth weight infants, the oral administration of *Bifidobacterium breve* M-16V allowed its gut colonization for several weeks post-administration and appeared to improve gut microbiota formation. In addition, bifidobacteria isolated from dogs were characterized *in vitro* and *in vivo* for potential use to support canine health (Jang et al.).

The period immediately following birth is crucial for the appropriate development of the gut microbiota and infant development (Turroni et al., 2020). In recent years, scientific efforts have tried to explain how commensal bacteria are established in the infant gut. Breast milk is a factor driving the transfer of functionally important commensal bacteria from mother to infant, specially *Bifidobacterium* species that efficiently colonize the infant gut. Yan et al. explored the co-occurrence of *Bifidobacterium* phylotypes in mother–breast milk–infant triads. The authors describe that the *gro*EL gene is an effective target for in depth resolution of *Bifidobacterium* communities. This approach allowed the assignment of *Bifidobacterium* phylotypes in mother–infant pairs.

To shed light on probiotic features and gut adaptation of bifidobacteria more mechanistic and functional analysis are needed through embracing different “omics” approaches in combination with novel/existing molecular tools, technologies and models. Members of the genus *Bifidobacterium* are notoriously recalcitrant to genetic manipulation due to presence of Restriction-Modification (R-M) systems. In this regard, a novel genetic tool for bifidobacterial targeted mutagenesis, based on a synthetic vector (pFREM28) lacking known *B. breve* R-M motifs, was described and validated by Hoedt et al.. This approach can be applied to design synthetic plasmids to target other genetically inaccessible bifidobacterial species. Additionally, an adapted plasmid expressing mCherry with OVA (pMG-mCherry-OVA) for bifidobacteria was developed to study bifidobacterial-host interactions in both *in vitro* and *in vivo* studies (Hickey et al.).

In summary, the integration of various approaches to determine the presence and functionality of bifidobacteria in the gut are crucial to improve our understanding of the complex interactions between the human (gut) and (health-promoting) bifidobacteria. Future probiotic interventions directed to modulate gut microbiota will lead to “next-generation” probiotic bifidobacterial strains in functional food and nutraceutical industries.

## Author Contributions

MET and DS wrote and edited the manuscript. All authors have made substantial contributions to the article and approved the manuscript for publication.

## Funding

DS and MET are members of APC Microbiome Ireland, which is a research center funded by the Science Foundation Ireland (SFI), through the Irish Government's National Development Plan. The authors and their work were supported by SFI (Grant SFI/12/RC/2273), Irish Research Council Postdoctoral Fellowship (GOIPD/2017/1302) and MSCA-IF (Grant number 898088). LR has received funding from the Spanish State Research Agency RTI2018-095021-J-I00 (funded by MCIU/AEI/FEDER, UE).

## Conflict of Interest

The authors declare that the research was conducted in the absence of any commercial or financial relationships that could be construed as a potential conflict of interest.

## Publisher's Note

All claims expressed in this article are solely those of the authors and do not necessarily represent those of their affiliated organizations, or those of the publisher, the editors and the reviewers. Any product that may be evaluated in this article, or claim that may be made by its manufacturer, is not guaranteed or endorsed by the publisher.
